# Diminished Peripheral CD29hi Cytotoxic CD4+ T Cells Are Associated With Deleterious Effects During SIV Infection

**DOI:** 10.3389/fimmu.2021.734871

**Published:** 2021-10-13

**Authors:** Omalla A. Olwenyi, Samuel D. Johnson, Kabita Pandey, Michellie Thurman, Arpan Acharya, Shilpa J. Buch, Howard S. Fox, Anthony T. Podany, Courtney V. Fletcher, Siddappa N. Byrareddy

**Affiliations:** ^1^ Department of Pharmacology and Experimental Neuroscience, University of Nebraska Medical Center, Omaha, NE, United States; ^2^ Department of Pathology and Microbiology, University of Nebraska Medical Center, Omaha, NE, United States; ^3^ Department of Neurological Sciences, University of Nebraska Medical Center, Omaha, NE, United States; ^4^ Antiviral Pharmacology Laboratory, Center for Drug Discovery, University of Nebraska Medical Center (UNMC), Omaha, NE, United States; ^5^ Department of Genetics, Cell Biology, and Anatomy, University of Nebraska Medical Center, Omaha, NE, United States; ^6^ Department of Biochemistry and Molecular Biology, University of Nebraska Medical Center, Omaha, NE, United States; ^7^ Division of Clinical Microbiology, Department of Laboratory Medicine, Karolinska Institute, Stockholm, Sweden

**Keywords:** CD4+ CTLs, SIV, morphine, reservoirs, CD29+ CD4+ T cells, biomarker

## Abstract

Cytotoxic CD4+ T cells (CD4+ CTLs) limit HIV pathogenesis, as evidenced in elite controllers (a subset of individuals who suppress the virus without the need for therapy). CD4+ CTLs have also been shown to kill HIV-infected macrophages. However, little is known about their contribution towards HIV persistence, how they are affected following exposure to immune modulators like morphine, and what factors maintain their frequencies and function. Further, the lack of robust markers to identify CD4+ CTLs in various animal models limits understanding of their role in HIV pathogenesis. We utilized various PBMC samples obtained from SIV infected and cART treated rhesus macaques exposed to morphine or saline and subjected to flow cytometry evaluations. Thereafter, we compared and correlated the expression of CD4+ CTL-specific markers to viral load and viral reservoir estimations in total CD4+ T cells. We found that CD29 could be reliably used as a marker to identify CD4+ CTLs in rhesus macaques since CD29hi CD4+ T cells secrete higher cytotoxic and proinflammatory cytokines following PMA/ionomycin or gag stimulation. In addition, this immune cell subset was depleted during untreated SIV infection. Strikingly, we also observed that early initiation of cART reconstitutes depleted CD29hi CD4+ T cells and restores their function. Furthermore, we noted that morphine exposure reduced the secretion of proinflammatory cytokines/cytotoxic molecules in CD29hi CD4+ T cells. Lastly, increased functionality of CD29hi CD4+ T cells as depicted by elevated levels of either IL-21 or granzyme B hi T Bet+ gag specific responses were linked to limiting the size of the replication-competent reservoir during cART treatment. Collectively, our data suggest that CD4+ CTLs are crucial in limiting SIV pathogenesis and persistence.

## Introduction

The proposed excision of latently infected HIV cells in a second person with HIV infection offers renewed optimism towards a cure that would usher an end to the AIDS crisis (1, 2). Although great strides have been undertaken, the cellular responses associated with sustaining persistence *versus* viral eradication remain poorly understood (3–5). HIV-infected individuals worldwide live different lifestyles stemming from varied cultural and sexual practices, co-exposures to other pathogens, and comorbid substance abuse not limited to the illicit use of drugs like morphine, cocaine, and heroin (6–11). Collectively, these behaviors differentially impact the body’s immune response, alter disease pathogenesis and need to be considered during the development of a universal HIV cure (6, 12).

The body’s immune system is built around the activities of CD4+ T cells (13). CD4+ T cells are a significant immune cell subset, which coordinates the immune system and also aid diverse immune (B, CD8^+^ T, and NK) cell function (14–16). In addition, they are crucial in generating prompt and protective memory responses against recalled pathogens (17, 18). CD4+ T cells are highly plastic and exist as numerous phenotypes like T helper (Th) 1, Th 2, and Th 17, cytotoxic (CD4+ CTL), T follicular helper (Tfh), and T regulatory (T regs) (19). These diverse CD4+ T cell phenotypes express different transcriptional profiles during health and disease, have distinct fates, and carry various functions, including immune regulation (CD+ T regs) (20). Beyond the simplistic provision of help, CD4+ T cells have also been shown to play other roles like regulation of immune responses (CD4+ T regs). They could also directly target infected cells through CD4+ CTLs (13). In supercentenarians, it was suggested that CD4+ CTLs were crucial in healthy aging, where they were found to be expanded while offering long-lasting protection (21).

The hallmark of HIV infection is the progressive loss of total CD4+ T cells and dysregulation of homeostasis, later culminating in acquired immune deficiency syndrome (AIDS) (22, 23). Early during infection, HIV causes massive gut damage accompanied by leakage of microbial biproducts into the periphery (24, 25). Synchronously, gut dysbiosis occurs as the landscape of the gut microbiome shifts towards more pro-inflammatory and pathogenic bacterial communities such as *Prevotella* and *Enterobacteriaceae* (26–28). As a result of microbial translocation and gut dysbiosis, chronic immune activation and systemic inflammation later ensue (29, 30).

Progressively, enhanced CD4+ T cell activation leads to immense CD4+ T cell loss by apoptosis and exaggerated viral cytolysis (31, 32). Following combined antiretroviral therapy (cART) initiation, the recovery of absolute CD4+ T-cell counts is often viewed as the benchmark for the immune system reconstitution (33, 34). However, ongoing dysregulation of cellular function limits the implementation of robust antiviral responses (35). Furthermore, specific CD4+ T-cell lineages like Tfh have been extensively reported to harbor viral reservoirs within the periphery and lymphoid tissues (36, 37). Although CD4+ T cells are targeted during virus replication and persistence, considerable evidence supports a crucial role of cytotoxic CD4+ T-cell phenotypes in HIV control and slowing disease progression (38, 39). During acute HIV infection, a robust HIV-specific CD4+ CTL response comprised of elevated granzyme A, interferon-gamma (IFN γ+), and CD40 ligand (CD40L) has been documented to lower viral load set points (40). Johnson et al. also made similar observations and noticed that CD4+ CTLs predominantly expressed perforin, granzyme B, and Eomes during acute HIV infection (38). Subsequently, HIV Nef-specific CD4+ CTLS has been documented to suppress viral replication in macrophages *ex-vivo* (41).

The lack of a specific CD4+ CTL surface biomarker with consensus across different animal models limits the proper follow-up and interrogation of these cells. Recently, studies by Johnson et al., and Phetsouphanh et al., together used CD57 to identify and track this cell phenotype in different HIV-1 infection conditions (38, 44). Despite this, the lack of cross-reactive antibodies raised against the CD57 carbohydrate epitope in non-human primate (NHP) models limits its use (45, 46). However, recently, in a pre-print article, Nicolet et al. suggested that CD29 enriches human CD4+ CTLs (47). Nonetheless, the utility of this biomarker as a surface marker for CD4+ CTLs remains to be evaluated in NHP models. Although various efforts have been undertaken to explore the effects of CD4+ CTL with HIV disease progression (39, 48), the impact of virus-specific functionality of this cell phenotype on the size of replication-competent reservoirs remains to be studied. Moreover, the modulation of this cell lineage following exposure to substance abuse such as morphine remains unevaluated.

To address some of above-mentioned questions, we utilized samples obtained from SIV-infected rhesus macaques that were exposed to either morphine or saline. Of these animals, a subset was also treated with cART to suppress viral replication. We found that enhanced CD29+ expression on the surface of CD4+ T cells could be used to identify CD4+ CTLs. Thus, CD29hi CD4+ T cells express higher cytotoxic molecules like CD107a and granzyme B plus proinflammatory cytokines like IFN-γ and TNF-α compared to their CD29lo counterparts. In addition, increased gag-specific secretion of cytokines like IL-21 and granzyme B was accompanied by smaller sizes of replication-competent viral reservoirs suggesting that CD4+ CTLs contribute towards limiting SIV persistence in cART-treated rhesus macaques.

## Materials And Methods

### Ethics Statement

This study was approved by the University of Nebraska Medical Center (UNMC) Institutional Animal Care and Use Committee (IACUC) as designated by assigned protocol numbers. For this study, we used: (1) 16-073-07-FC named “The Effect of cART and Drugs of Abuse and the Establishment of CNS Viral Reservoirs”. (2) 15-113-01-FC referred to as “The Combinatorial effects of Opiates and Promoter Variant Strains of HIV-1 subtype C on Neuropathogenesis and Latency”.

### Rhesus Macaques Used for This Study

For protocol 16-073-07-FC, 6 animals were escalated to 6mg/kg twice daily injections of morphine alongside five rhesus macaques exposed to saline (controls). Following this, all the animals were infected with 200 TCID_50_ of SIVmac251 and later treated with a cART regimen comprising of 40 mg/ml emtricitabine (FTC), 20 mg/ml tenofovir (TFV), and 2.5 mg/ml dolutegravir (DTG). Alternatively, protocol 15-113-01-FC involved 8 rhesus macaques separately exposed to equivalent doses of either morphine (n = 4) or saline (n = 4). Similarly, this was followed with SIVmac251infection at an equivalent dose. However, no subsequent cART was administered. All animal characteristics and treatment regimen details were previously described (10). The experimental schema utilized for the study included a total of 19 rhesus macaques. Briefly, in the untreated group of eight animals, 6-mg/kg intramuscular (i.m.) injections of morphine were administered twice daily for 2 weeks to four rhesus macaques. Then, continued morphine dosing was maintained for 7 weeks while the remaining four animals received normal saline (control group). After 9 weeks, 200 TCID_50_ of SIVmac251 was intravenously administered to the rhesus macaques and continued with morphine/saline treatments until the end of the study. (B) In the separate group of 11 animals exposed to cART, six were given intramuscular morphine for a total of 9 weeks while five received equal doses of saline (control group). After 9 weeks, 200 TCID_50_ of SIVmac251 was intravenously administered to all animals. Five weeks later, cART was initiated and given daily at 1 ml/kg body weight up to the termination end of the study. Saline was given to the controls at a similar dose. Lastly, to evaluate the effects of cART on CD29hi CD4+ T cells, we utilized eight rhesus macaques exposed to either four drugs (FTC, tenofovir alafenamide (TAF), DTG and maraviroc (MVC); n = 4) or two drugs (DTG and FTC; n = 4) that were treated four weeks post SIV inoculation (protocol #16-001-02-FC, Assessment of Antiretroviral Pharmacology in Lymphoid Tissues using the SIV Macaque Model).

### Isolation and Cryopreservation of PBMCs

Femoral blood, collected in K2-EDTA vacutainer tubes (BD, 367841), was layered above Lymphoprep™ Density Gradient Medium from STEMCELL Technologies following plasma separation. PBMCs were then later isolated using the density gradient centrifugal separation method described previously (49).

### Phenotype Analysis of Immune Cells (Flow Cytometry)

Cryopreserved PBMCs processed from the blood of rhesus macaques included in this study were utilized for flow cytometry. Post-thaw viabilities of greater than 80% were considered for further experimentation. Briefly, this involved a brief 12-18h rest, and surface staining protocols proceeded at this step. First, Zombie Aqua fixable viability dye was added to discriminate dead cells. After that, all surface markers included in the lineage and integrin/chemokine receptor panels (**Supplementary Table 1**) were added at previously titrated volumes. Fixation was then performed using 2% PFA and samples acquired using the Becton Dickson Fortessa X450 flow cytometer. Intracellular staining protocols involved stimulation of SIV mac239 gag peptide mix spanning 15-mers with 11-aa overlap (ARP-6204) obtained through the NIH HIV Reagent Program, Division of AIDS, NIAID, NIH: Peptide Array, Simian Immunodeficiency Virus (SIV)mac239 Gag Protein, ARP-6204, contributed by DAIDS/NIAID) and PMA (Phorbol myristate acetate)/ionomycin (20 ng/ml and 0.5 μg/ml) respectively dissolved in 10% complete media. Simultaneously, Golgi plug containing Brefeldin A and the Golgi transport inhibitor monensin was added to inhibit the release of cytokines and cytotoxic granules into the supernatant. After 6 hours of stimulation, 20 mM EDTA solution was added. After that, live/dead exclusion was performed, and surface staining for CD29 PercpCy5.5 was carried out. For the cytotoxicity panel (**Supplementary Table 1**) involving Eomes and T-bet transcription factors, the fix/perm solution (Tonbo Biosciences) was used for fixation and permeabilization. The remaining antibody cocktail was dissolved in FoxP3 transcription factor buffer (Tonbo biosciences) and added following permeabilization. Alternatively, in the cytokine secretion panel (**Supplementary Table 1**), 2% PFA was added for fixation, followed by permeabilization using BD perm wash buffer. Then, cells were incubated with a previously prepared cocktail of antibodies. Following incubation and later washes, the acquisition was performed on the Becton Dickson Fortessa X450 flow cytometer, and analysis was carried out using Flowjo version 10.6 (Trees Star Inc., Ashland, Oregon, USA). For ICS quantification, all values were reported following background subtraction.

### Quantification of Plasma Viral Load in Peripheral Blood

Quantitative real-time PCR (qRT-PCR) was utilized to estimate levels of SIV RNA in EDTA plasma (50). Briefly, RNA was extracted from EDTA plasma using a QIA amp viral RNA mini kit (Qiagen, Germantown, MD, USA; Cat no: 52906). Additional information on primers and probes with details of PCR conditions were described previously (10).

### Quantitative Viral Outgrowth Assay (QVOA)

CD4+ T cells were enriched from PBMCs using the non-human primate microbead CD4+ T cell isolation kit, (STEMCELL Technologies Inc, Seattle, WA, USA) and later resuspended in complete media (RPMI1640, 10% FBS, 2 mM glutamine, Penstrep (100 U/mL penicillin and 100 μg/mL streptomycin)), 10 U/mL IL-2 and 300 nM efavirenz (EFV; NIH AIDS Reagent Program). The resultant purified CD4+ T cells were then co-stimulated with CD3/CD28 beads (Dynabeads, Life Technologies, Waltham, MA, USA) and 10-fold limiting dilutions ranging from 10^2^ to 10^5^ cells/mL were generated. Following 72 h of incubation, 10^5^ CEMx174 cells were added to aid in viral expansion. This time point was noted as day 0 of incubation and was later prospectively followed for 21 days. SIV RNA measures were then performed using qRT-PCR. Levels of replication-competent viruses were denoted as infectious units per million (IUPM) and were estimated using the IUPMStats v1.0 infection frequency calculator (10, 51).

### Statistical Analysis

Prism V9.0 (GraphPad Software) was used for Spearman’s rank correlation and paired non-parametric tests with the aid of the Wilcoxon test. In addition, comparisons between morphine and saline groups were also performed using the Mann Whitney U test. Using R version 3.4.3, a correlation matrix was generated following multiple correlations. Resultant P values were then corrected for type 1 error with the aid of Holm’s correction. Lastly, following Boolean gating in FlowJo, combinatorial expression of multiple cytokines was evaluated and graphed using SPICE (“Simplified Presentation of Incredibly Complex Evaluations”) (52).

## Results

### Loss of CD29hi CD4+ T Cells Is Positively Interrelated to Declining Immune Status and Increasing Viral Loads During SIV Infection

**Figure 1:**
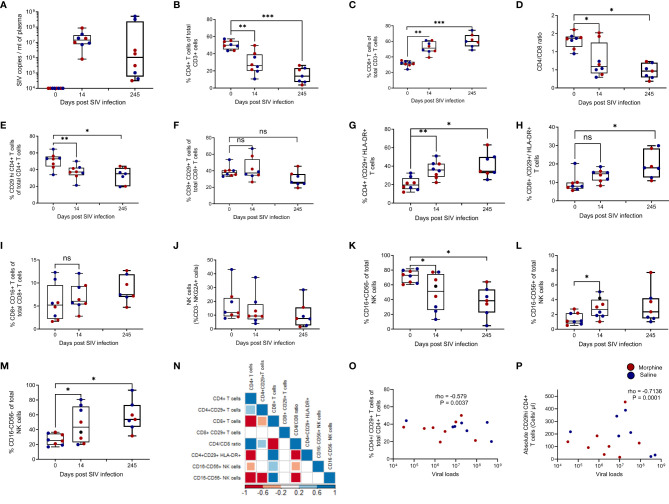
Loss of CD29 hi CD4+ T cells are associated with increased viral loads, declining immune status, and dysregulated NK cell subsets in SIV infected rhesus macaques exposed to either morphine or saline. Viral loads at baseline (day 0), acute (day 14), and chronic (day 245) during untreated SIV infection **(A)**. Changes in percent CD4+ T cells **(B)**, percent CD8+ T cells **(C)**, CD4/CD8 ratio **(D)**, percent CD29+ CD4+ T cells **(E)**, percent CD29+ CD8+ T cells **(F)**, percent CD29+/CD4+/HLA-DR+ **(G)**, percent CD29+/CD8+/HLA-DR+ **(H)** and CD8+/CD16+ T cells **(I)** at the three studied time points (day 0, day 14 and day 245) post infection. Evaluating NK cells and comprising subsets that include changes in total NK cells **(J)**, CD16+CD56- NK cells **(K)**, CD16-CD56+ NK cells **(L)**, and CD16-CD56- NK cells **(M)** at day 0, day 14, and day 245 post infection. **(N)** Correlogram showing correlations between various immune cell subsets and percent CD29+ CD4+ T cells.. Relationship between either **(O)** percent CD29+ CD4+ T cells or **(P)** absolute CD29+ CD4+ T cells and viral load. * shows p < 0.05, ** indicates p < 0.01 while *** denotes p < 0.001 resulting from Wilcoxon matched pairs signed rank tests between paired groups of the different comparisons. ns represents non significant. Per visit comparisons between morphine and saline were also performed using the Mann Whitney U test (non- significant).

To gain insight into cellular and virological interactions of the peripheral immune system, changes in CD29hi CD4+ T cells alongside viral load and various immune cell subsets during baseline, acute (Day 14), and chronic (Day 245) phases of SIV disease progression (**Supplementary Figure 1** **)**. Following SIV infection, viral load levels peaked during the acute phase and were maintained through the chronic time point (**Figure 1A**). Compared to baseline, there was a progressive loss in %CD4+ T cell frequencies across the acute phase of infection (P = 0.0078) and the latter chronic phase of infection (P = 0.0003) (**Figure 1B**). Subsequently, this was accompanied by an expansion of CD8+ T cells during both the acute (P = 0.0078) and chronic (P = 0.0002) phases of untreated SIV infection (**Figure 1C**). Unsurprisingly, there was a progressive loss in the CD4/CD8 ratio across the acute (P = 0.0156) and chronic (P = 0.0156) phases of infection (**Figure 1D**). The frequencies of %CD29hi CD4+ T cells were reduced during the acute (P = 0.0469) and chronic (P = 0.0078) phases of untreated infection (**Figure 1E**). Similar trends were observed with absolute CD29hi CD4+ T cells (**Supplementary Figure 2A**) and absolute CD4+ T cells (**Supplementary Figure 2B**), while absolute CD8+ T cells were elevated during SIV progression (**Supplementary Figure 2C**). Remarkably, %CD8+/CD29+ T cells remained stabilized during the acute and chronic phases of untreated SIV infection despite the concurrent expansion of CD8+ T cells (**Figure 1F**). The percent of both CD29hi CD4+ T cells and CD29+/CD8+ T cells exhibited an increase in activation shown by increased expression of HLA-DR (P < 0.05) (**Figures 1G** **, H** **).** No changes were observed in innate-like CD8+ T cells that express CD16 (**Figure 1I** **)**. A progressive non-significant reduction of NK cells occurs through the acute to chronic phases of untreated SIV infection (**Figure 1J** **)**. However, NK cell subset analysis revealed that there was a reduction in %CD16+CD56- NK cells, (P = 0.024) (**Figure 1K** **)** together with concurrent expansions of %CD16-CD56+ NK cells, (P = 0.039) (**Figure 1L** **)** and %CD16-CD56- NK cells (P = 0.039) that are later maintained during chronic infection (**Figure 1M** **)**. Multiple Spearman rank correlation analysis revealed that reduced %CD29hi CD4+ T cells were positively associated with expanding %CD8+ T cells (rho = -0.532, P = 0.0044), CD16-CD56+ NK cells (rho = -0.647, P = 0.0006) while a positive correlation was seen with improved immune status (CD4/CD8 ratio) (rho = 0.42, P = 0.044). Additionally, negative associations were noticed with CD16-CD56- NK cells (rho = -0.7278374) and CD16-CD56+ NK cells (rho = -0.3928776), (all P < 0.05) (**Figure 1N** **)**. Furthermore, CD16+CD56- NK cells were positively correlated with absolute CD29hi CD4+ T cells (rho = 0.7510 and P = 0.00001) and absolute CD4+ T cells (rho = 0.7206 and P = 0.0002) respectively (**Supplementary Figures 2D, G**). In turn, CD16-CD56+ NK cells were associated with loss of CD29 hi CD4+ T cells (**Supplementary Figure 2E** **)** and declining absolute CD4+ T cells (**Supplementary Figure 2H** **)** during SIV disease progression. Similarly, CD16-CD56- NK cells were also associated with loss of CD29hi CD4+ T cells (**Supplementary Figure 2F** **)** and reduced absolute CD4+ T cells (**Supplementary Figure 2I** **)** during infection. Lastly, percent and absolute CD29hi CD4+ T cells were seen to negatively associate with viral loads [(rho = -0.57 and P = 0.0037) and (rho = -0.7136 and P = 0.0001)] (**Figures 1O** **, P** **)**.

### Early Initiation of cART (4 Weeks Post-Infection) Supports Percent CD29hi CD4+ T Cells Restoration and Rescued Polyfunctionality Within the Periphery

**Figure 2:**
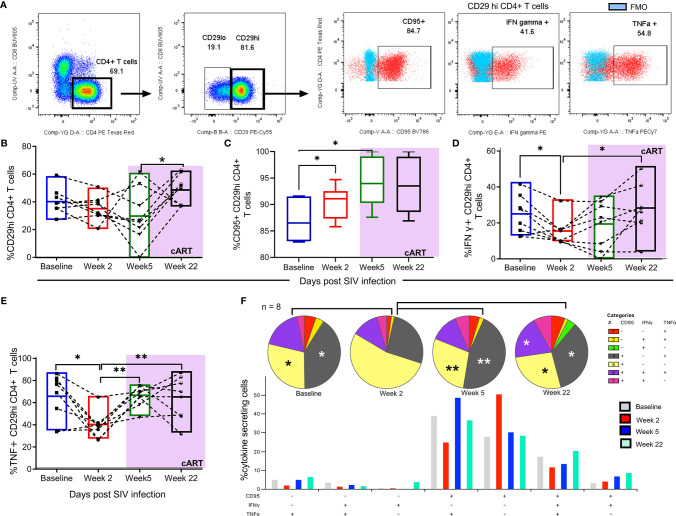
Early cART initiation (4 weeks) restores the loss of CD29hi CD4+ T cells and restores polyfunctionality. **(A)** Representative gating used to obtain CD4+ CD29 hi T cells used for changes in cytokine secretion at different stages of SIV progression. Briefly, CD4+ T cells were obtained from gated CD3+ T cells/lymphocytes/live cells/singlets/Time vs FSC-A populations. Thereafter, CD29 hi populations were then gated out from CD4+ T cells and the extent of CD95 expression and IFN-γ/TNF-α secretion. Following this, the changes of **(B)** %CD29hi CD4+ T cells **(C)** %CD95+ CD29hi CD4+ T cells **(D)** IFN-γ+ CD29 hi CD4+ T cells **(E)** TNF-α+ CD29hi CD4+ T cells evaluated at baseline, week 2, week 7 and week 22 post SIV infection. **(F)** Polyfunctionality profiles of CD29hi CD4+ T cells stimulated with PMA/ionomycin. Included pie charts/bar graphs represent time course changes in CD29hi poly functionality based on IFN-γ, TNF-α and CD95 expression (n = 8). Bar graphs show the median percent cytokine expressing cells. All paired comparisons across the different timepoints were performed using the non-parametric Wilcoxon test. * shows p < 0.05 while ** indicates p < 0.01.

In a separate set of rhesus macaques (n = 8), early cART was initiated to test whether depleted peripheral CD29hi CD4+ T cells could be reconstituted. In addition, retrospective time course evaluations were carried out to evaluate how different stages of SIV disease progression and therapy affect the functionality of this cell subset. Representative gating of CD29hi CD4+ T cell subset reveals CD95+, IFN-γ+ and TNF α+ cytokine secretion following PMA/ionomycin stimulation (**Figures 2A** and **Supplementary Figure 3** **)**. Changes in %CD29hi CD4+ T cells, %CD95 CD29hi CD4+ T cells, IFN-γ+ CD29hi CD4+ T cells and TNF-α + CD29hi CD4+ T cells showed that six weeks post untreated SIV infection, a progressive loss CD29hi CD4+ T cells in conjunction with inflammatory cytokines occurred. CD95+ cell expression was elevated during acute SIV infection and remained steadily maintained post-therapy. In contrast, IFN-γ+ and TNF-α + cytokine levels diminished before ART but recovered after therapy (**Figures 2B** **, E** **)**. Finally, early initiation of cART restored lost polyfunctionality of CD29hi CD4+ T cells (% (CD95+, IFN-γ+ and TNF α+) and [% CD95+, IFN-γ- and TNF α+)], (**Figures 2F** **)**.

### CD29hi CD4+ T Cells Express Elevated Levels of Pro-Inflammatory and Cytolytic Mediators in Rhesus Macaques Exposed to cART and Morphine During Chronic Infection

**Figure 3:**
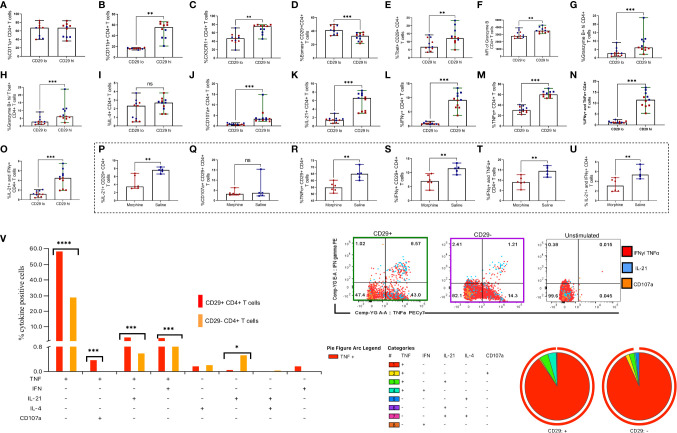
CD29hi CD4+ T cells identify cytotoxic CD4+ T cells (CD4+ CTLs) with superior cytotoxicity, expression of inflammatory surface molecules and cytokines following stimulation with PMA/ionomycin in cART treated rhesus macaques exposed to saline or morphine during chronic infection. The expression percent CD11a **(A)**, percent CD11b **(B)**, the chemokine receptor CX3CR1 **(C)**, percent Eomes **(D)** and percent T bet **(E)** was evaluated and compared between CD29lo vs. CD29hi T cells. Similarly, cytotoxicity was evaluated based on percent granzyme B **(F)**, percent Granzyme B hi **(G)**, and Granzyme B hi and T bet co-expression **(H)**. In addition, the expression of percent IL-4 **(I)**, percent CD107a **(J)**, percent IL-21 *(K)*, percent IFN-γ **(L)**, percent TNF-α **(M)**, dual co-expression of IFN-γ and TNF-α **(N)** and IL-21 and IFN-γ co-expression **(O)** was evaluated. The changes in the secretion of cytokines and cytotoxic molecules IL-21 **(P)**, CD107a **(Q)**, TNF-α **(R)**, IFN-γ **(S)**, IFN-γ and TNF-α **(T)** and IL-21 and IFN-γ **(U)** within CD29 hi CD4+ T cells following morphine exposure were evaluated using ICS. Representative gating of CD29 hi *versus* CD29 lo cells expressing multiple cytokines such as IL-21, CD107a, TNF-α and IFN-γ **(V)**. Polyfunctional profile of CD29hi vs CD29lo CD4+ T cells responding to PMA/ionomycin. (I) Representative manual gating of CD29hi vs CD29lo CD4+ T cells used to analyze polyfunctionality. (II) and (III) Pie charts and the bar plots show the frequency of CD29+ or CD29- CD4+ T cells expressing different combinations of cytokines (TNF-α, IFN-γ, IL-21, IL-4 and CD107a) in n=11 rhesus macaques. Paired comparisons between CD29lo and CD29hi CD4+ T cells were performed using the non-parametric Wilcoxon test. Comparisons between morphine and saline were also performed in CD29+ CD4+ T cells using the Mann Whitney U test. For SPICE analyses, pie charts showing cytokine secretion patterns together with arc plots show the predominant cytokine being secreted. Bar graphs denote median cytokine expressing cells. Simultaneously, the extent of cytokine polyfunctionality was compared within CD29lo vs CD29 hi CD4+ T cell subsets with aid of the Wilcoxon test. For all comparisons, * shows p < 0.05, ** indicates p < 0.01 while *** denotes p < 0.001. ns represents non significant. Red dots denote morphine treated while blue dots represent rhesus macaques exposed to saline.

Upon noticing that CD29hi CTLs were depleted during progressive untreated SIV infection, we next turned our efforts to characterizing these cells. Utilizing PBMCs collected from cART treated rhesus macaques, we assessed the extent to which CD4+ CD29hi T cells express diverse integrins (CD11a and CD11b) and the chemokine receptor CX3CR1 (**Supplementary Figure 4**). In addition, after PMA/ionomycin stimulation, we assessed transcription factors T bet and Eomes, the cytotoxic mediators granzyme B and CD107a, IL-21, T helper (Th) 1 cytokines TNF-α and IFN-γ plus the Th2 cytokine IL-4 (**Supplementary Figure 5**). Surprisingly, there were no significant differences between CD11a expression in CD29hi vs. CD29lo CD4+ T cell subsets (**Figure 3A** **)**. However, we noticed a three-fold increment of surface CD11b expression on CD29hi *versus* CD29lo CD4+ T cells (P = 0.0020) (**Figure 3B** **)**. Similarly, we observed a close to two-fold elevation in the expression of CX3CR1 in CD29hi CD4+ T cells (P = 0.0010) **(** **Figure 3C** **).** Unexpectedly, the CD29hi CD4+ T cell subset had lower levels of Eomes (P = 0.0020) **(** **Figure 3D** **)** while expressing over two-fold levels of T bet in comparison to the CD29lo CD4+ T cell subset (P = 0.0020) **(** **Figure 3E** **)**. Moreover, elevated granzyme B expression was observed in the CD29hi CD4+ T cell subset *versus* the CD29lo CD4+ T cell subset **(** **Figure 3F** **)**. Similarly, the level of granzyme B+ hi expression was elevated in the CD29hi CD4+ T cell subset (P = 0.0010) **(** **Figure 3G** **),** which remained augmented even after testing for co-expression of granzyme B hi and Tbet+ (P = 0.0010) **(** **Figure 3H** **)**. The level of IL-4 was similar between CD29hi vs CD29lo CD4+ T cells **(** **Figure 3I** **)** whilst elevated expression of diverse cytokines such as: CD107a (P = 0.0010) **(** **Figure 3J** **)**, IL-21 (P = 0.0010) **(** **Figure 3K** **),** IFN-γ (P = 0.0010) **(** **Figure 3L** **)** and TNF-α (P = 0.0010) **(** **Figure 3M** **)** were observed. Similarly, there were higher frequencies of % IFN-γ+ and TNF-α + co-expressing CD29hi CD4+ T cells in comparison to CD29lo CD4+ T cells (P = 0.0087) (**Figure 3N**). Likewise, IL-21+ and IFN γ+ co-expressing cells are elevated in CD29hi CD4+ T cells in comparison to CD29lo CD4+ T cells (P = 0.0010) **(** **Figure 3O** **)**. Next, we observed that morphine causes downregulation of cytokine secretion ranging from IL-21, TNF-α, IFN-γ, IFN-γ, and TNF-α dual positive plus IL-21 and IFN-γ co-expressing CD29hi CD4+ T cells (all P < 0.01) (**Figures 3P–U**). Morphine treatment was also observed to similarly downmodulate the expression of inflammatory cytokines and cytotoxic molecules in the CD29lo CD4+ T cell subset (**Supplementary Figure 6**). By evaluating polyfunctionality of CD29hi CD4+ T cells revealed that the majority of cytokine-producing cells were mainly TNF-α + cells and demonstrated greater frequencies of multiple cytokines secreting cells such as combinations with TNF-α + and CD107a cells, TNF-α + and IL-21 and TNF-α + and IFN-γ+ co-expressing cells, (all P <0.05) (**Figure 3V**).

### CD29hi Gag Specific CD4+ T Cells Also Express Elevated Levels of Pro-Inflammatory and Cytolytic Mediators in Rhesus Macaques Exposed to cART and Morphine

**Figure 4:**
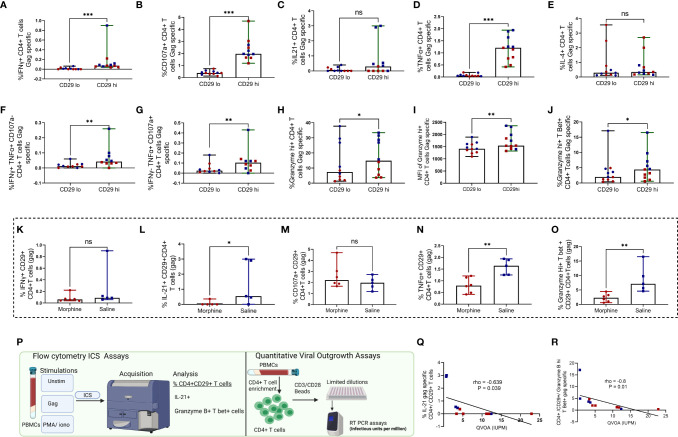
CD29 hi CD4+ T cells express elevated inflammatory cytokines and cytotoxic molecules following stimulation with gag peptides in cART treated rhesus macaques exposed to saline or morphine during chronic infection. Comparative expression of inflammatory cytokines and cytotoxic molecules between CD29 hi vs CD29 lo CD4+ T cells was performed as depicted by percent IFN-γ **(A)**, percent CD107a **(B)**, percent IL21 **(C)**, percent TNF-α **(D)**, percent IL-4 **(E)**, percent IFN-γ+ TNF-α + and CD107a- **(F)**, percent IFN-γ- TNF-α + and CD107a+ **(G)**, Granzyme B hi **(H)**, MFI of Granzyme B+ hi **(I)**, Granzyme B+ hi T bet+ **(J)** gag specific CD4+ T cells. Immune modulation by morphine on CD29 hi CD4+ CTLS was analyzed for the expression of various cytokines such as percent IFN-γ **(K)** percent IL21 **(L)** percent CD107a **(M)**, percent TNF-α **(N)**, percent Granzyme B+ hi T bet+ cells **(O)**. Scheme for correlation analysis between gag specific IL-21 or Granzyme B hi T bet+ CD29 hi CD4+ T cells and QVOA measures of replication competent reservoirs in total CD4+ T cells **(P)**. Correlation between gag-specific IL-21+ CD29hi CD4+ T cell frequencies and QVOA **(Q)**. Correlation between gag specific Granzyme B+ hi T bet+ CD29hi CD4+ T cell frequencies and QVOA **(R)**. Paired comparisons between CD29 lo and CD29 hi CD4+ T cells were performed using the non-parametric Wilcoxon test. Comparisons between morphine and saline were also performed in CD29+ CD4+ T cells using the Mann Whitney U test. For all comparisons, * shows p < 0.05, ** indicates p < 0.01 while *** denotes p < 0.001. ns signifies non significant. Red dots denote morphine treated while blue dots represent rhesus macaques exposed to saline.

In synchrony with earlier observations seen in **Figure 2**, CD29 hi CD4+ T cells expressed higher levels of IFN-γ (**Figure 4A**), CD107a (**Figure 4B**), IL-21 (**Figure 4C**), TNF-α (**Figure 4D**) (all P<0.05) (**Supplementary Figure 6**). The same trend was seen with TNF-α+ and CD107a- co-expressing cells (**Figure 4F**), IFN-γ-, TNF-α+ and CD107a+ co-expressing cells (**Figure 4G**), percent and MFI granzyme B hi cytolytic molecules (**Figures 4H, I**) and granzyme B hi and T Bet+ co-expressing cells (**Figure 4J**) in comparison to CD29lo CD4+ T cells, (all P<0.05). Notably, no significant differences were observed with levels of IL-4 (**Figure 4E**), (P > 0.05). In congruence with earlier observations noticed, morphine similarly led to downregulation of various gag-specific cytokines such as IL-21, TNF-α, and granzyme B hi T Bet+ in CD29hi CD4+ T cells (all P < 0.05) **(** **Figures 4K** **–O** **)**. Next, we investigated whether there was a relationship between either IL-21 or granzyme B hi T Bet+ gag specific release on CD29hi CD4+ T cells with the size of the replication-competent viral reservoir as measured by quantitative viral outgrowth assay (QVOA) **(** **Figure 4P** **).** Negative correlations were observed between QVOA and either IL-21 gag specific CD4+/CD29+T cells or granzyme B hi T bet+ gag specific CD29 hi CD4+ T cells. The association between granzyme B hi T Bet+ CD29hi CD4+ (**Figures 4Q** **, R**).

## Discussion

CD4+ CTLs are crucial in limiting the pathogenesis and mediating protection against HIV/SIV infection. It was previously documented that elite controllers, a unique group of individuals capable of suppressing HIV without therapy, possess greater frequencies of circulating virus-specific CD4+ CTLs (44). Remarkedly, the development of CD4+ CTL escape SIV mutants is accompanied by loss of elite control in rhesus macaques (53). Thus, there is no surprise that declining immune status occurred simultaneously with the loss of proposed CD4+ CTLs and increased viral loads suggesting this cell subset is crucial in limiting HIV pathogenesis.

Similarly, the loss of HIV-specific CD4+ CTL function has been reported in chronic progressors (44). Predictably, during progressive infection, CD29hi CD4+ T cells exhibited increased HLA-DR activation levels, hinting that this cell phenotype could be targeted and depleted by the virus (54). Furthermore, the expansion of cytokine secreting (CD16-CD56+) and double negative CD16-CD56- NK cells are crucial for limiting viremia post-peak viral loads (55, 56). However, the loss of CD16 expression on prevailing NK cell subsets followed by the decrease of both CD29hi CD4+ T cells and absolute CD4+ T cells further highlights SIV-induced NK cell and CD4+ CTL dysfunction during disease progression (57).

During our time course studies, our observation that timely initiation of therapy reconstitutes peripheral CD29hi CD4+ T cells and restores IFN-γ, TNF-α, and CD95 polyfunctionality, in turn, supports findings from other groups who have reported that early ART preserves T cell function (58, 59). Following gag and PMA/ionomycin stimulation, CD29hi CD4+ T cells display cytotoxic properties through elevated secretion of proinflammatory cytokines such as IFN-γ, TNF-α, and IL-21 or cytolytic molecules such as granzyme and CD107a (60). In turn, increased expression of T bet dually favors IFN-γ and TNF-α secretion while supporting cytotoxicity by binding to downstream promoters of granzyme B and perforin genes (61).

Similarly, augmented expression of CX3CR1 also tends to favor CD4+ CTL transmigration into inflamed tissue, as exemplified in Dengue, where virus-specific CX3CR1 CD4+CTLs mediate protective cytotoxicity (62). Similarly, Chen et al. showed that a cytomegalovirus-driven CD57hi CD4+ CTL phenotype uses CX3CR1 to facilitate vascular trafficking by attaching to the fractalkine receptor on the endothelium during HIV infection (63).

Since CD29hi CD4+ T cells exhibit greater polyfunctionality as demonstrated by a distinct functional profile typified by elevated secretion of multiple inflammatory cytokines and cytotoxic molecules, they could serve as robust antiviral mediators that directly target virus-infected cells (59). In agreement with this, we noted that increased *ex-vivo* secretion of IL-21 in response to gag was negatively associated with the size of the replication-competent viral reservoir within the periphery. In a separate study, IL-21 secretion by CD4+ T cells was noted to support CD8+ cytotoxic T cell responses in chronic progressors, even during the late-stage infection, leading to diminished virus replication (64). Furthermore, therapeutic administration of IL-21 has been documented to reduce the size of viral reservoirs in rhesus macaques (64, 65).

The association between granzyme B hi T Bet+ CD29hi CD4+ T cells and diminished replication-competent viral reservoirs mirrors earlier findings by a separate study group found that HIV specific granzyme B and not IFN-g CD8 CTLs were linked to reduced HIV reservoirs during acute HIV infection (42). In addition, increased inflammation, as noted by elevated levels of pro inflammatory cytokines such as IL-18 (data not shown), has been linked to development of AIDS (43, 66). The observation that elevated CD29hi CD4+ CTLs were negatively associated with proinflammatory plasma IL-18 during untreated SIV infection, data not shown, corroborates the importance of these cells in fostering better disease outcomes.

The observation that morphine downmodulated the secretion of virus-specific proinflammatory cytokines and the release of granzyme B by CD4+ CD29hi CTLs coincides with several reports that suggest that this drug inhibits CD4+ T cell release of Th1 cytokines (IFN γ, TNF-α) while causing a shift towards Th2 phenotypes (67, 68). However, future studies are needed to evaluate the mechanisms by which morphine causes immune modulation of this cell subset. Further, larger sample sizes and studies using sorted CD29hi CD4+ CTLs are necessary for further validation of the contribution of this cell phenotype towards targeting persistence.

Additional future strategies aimed at improving the CD4+ CTL functionality, such as utilizing chimeric engineering strategies could reinvigorate CD4+ CTLs fostering improved disease outcomes (69, 70). Further, the unexpected transcription profile of lower Eomes expression in CD29hi CD4+ T cells while harnessing augmented levels of Tbet warrants further research in understanding how regulation of transcription factors affects CD4+ CTL functionality and differentiation. In their CD8+ T cell counterparts, Smith et al., revealed that cells expressing Tbet hi and Eomes lo profiles are more efficient in recognizing major histocompatibility complex (MHC) peptides (71). Finally, there is a need to track changes in this cell lineage in body compartments such as lymph nodes, central nervous system, and the gut that provide safe niches for low-level virus replication, evolution, and persistence following effective cART (72).

In conclusion, we demonstrate that CD29 could be reliably used to identify CD4+ CTLs in rhesus macaques. Furthermore, CD29hi CD4+ CTLs are beneficial towards limiting SIV pathogenesis by limiting virus replication and secreting crucial cytokines associated with reducing the size of the viral reservoir within the periphery.

## Data Availability Statement

The original contributions presented in the study are included in the article/**Supplementary Material**. Further inquiries can be directed to the corresponding author.

## Ethics Statement

The animal study was reviewed and approved by University of Nebraska Medical Center (UNMC) Institutional Animal Care and Use Committee (IACUC).

## Author Contributions

OO and SNB designed the conceptual framework of the study and analyzed the data. OO performed all the flow cytometry experiments, wrote the initial draft of the manuscript. SJ, KP, MT, and AA carried out virological assays. SJB, HF, AP, CF, and SNB supported fundraising for the animal studies and edited the manuscript. All authors contributed to the article and approved the submitted version.

## Funding

This study was supported by NIH grants R01DA043164, P30MH062261 to SNB, SJB, and HF, R01DA041751 to SNB and SJB, and R01AI124965 to CF. The funders had no role in designing the study or in the interpretation of the results.

## Conflict of Interest

The authors declare that the research was conducted in the absence of any commercial or financial relationships that could be construed as a potential conflict of interest.

## Publisher’s Note

All claims expressed in this article are solely those of the authors and do not necessarily represent those of their affiliated organizations, or those of the publisher, the editors and the reviewers. Any product that may be evaluated in this article, or claim that may be made by its manufacturer, is not guaranteed or endorsed by the publisher.

## Supplementary Material

Supplementary Figure 1 Longitudinal Changes In Diverse Lymphocyte Subsets During Progressive Untreated Siv Infection. Gating strategy based on a representative sample used for delineation of lymphocyte subpopulations within PBMCs obtained from untreated SIV rhesus macaques. Lymphocytes were gated out based on size and complexity (FSC-a vs. SSC-a). NKG2A vs. CD3+ was used to exclude CD3+ T cells, NKT cells, and NK cells. CD3+ T cells were later differentiated based on CD4+ and CD8+ markers and expression of CD29 was used to mark cytotoxicity in either subset. HLA-DR levels were then used to identify activation levels in either CD4+ or CD8+ T cells. MAIT cells were also studied based on CD161 and Vα7.2Jα co-expression. CD16+ CD8+ T cells (innate like CD8+ T cells) were also evaluated. NK cell subsets based on CD16 and CD56 expression were also studied for maturity based on CD27 and activation based on HLA-DR expression.

Supplementary Figure 2 Relationship between absolute t cell subsets and percent frequnencies of nk cell subsets during untreated siv disease progression. Changes in absolute: **(A)** CD29hi CD4+ T cells **(B)** CD4+ T cells **(C)** CD8+ T cells during baseline (day 0), day 14 and day 245 past SIV infection. Correlation between absolute CD29hi CD4+ T cells and: **(D)** %CD16+CD56- NK cells **(E)** %CD16-CD56+ NK cells **(F)** %CD16-CD56- NK cells. Additional correlations were also performed that included the association between absolute CD4+ T cells and: **(G)** %CD16+CD56- NK cells **(H)** %CD16-CD56+ NK cells **(I)** %CD16-CD56- NK cells. For **(A-C)**, ** indicates p < 0.001 while *** denotes p < 0.0001 resulting from Wilcoxon matched pairs signed rank tests between paired groups of the different comparisons. Per visit comparisons between morphine and saline were also performed using the Mann Whitney U test (non- significant).

Supplementary Figure 3 Representative Gating Of Time Course Changes In Cd29hi Cd4+ T Cells Across The Various Studied Time Points (Baseline, Week 2, Week 7 And Week 22). Representative gating for rhesus macaque CF42 indicating changes in CD95 expression, IFN-γ and TNF-α secretion across CD29hi CD4+ T cells within the studied time points. FMO controls also included for the accurate placement of gates.

Supplementary Figure 4 Determination Of Integrin (Cd11a And Cd11b) And Chemokine Receptor Expression (Cx3cr1) On Cd29hi Vs Cd29lo Cd4 T Cells During Art Treated Chronic Siv Infection. To achieve this, a time vs FSC-A gate was used to obtain a steady stream of cells during cell acquisition that is devoid of interruptions caused by micro bubbles, clogs or air. The FSC-A vs FSC-H gate and SSC-A vs SSC-H was used to obtain single cells. Dead cells were excluded using positive zombie aqua live dead staining. Lymphocytes were then segregated based on FSC-A vs SSC-A gate based on size and complexity and T cells later gated off using the CD3+ marker. CD4+ T cells were then further gated followed by the extent of their expression of CD29. Comparisons of CD29hi vs CD29lo CD4+ T cells were then carried out to evaluate the expression of CD11a and CD11B integrins together with the chemokine receptor CX3CR1. Fluorescent minus one (FMO) controls were included to set a background for gate placement.

Supplementary Figure 5 Evaluation Of Cytokine Expression In Cart Treated Siv Infected Macaues Exposed To Morphine Or Saline. Cells were excluded based on the FSC-a vs. time to obtain cells in a single stream. Doublets were excluded using FSC-A vs. FSC-H/SSC-A vs. SSC-H. Live cells were then excluded based on Live Dead Zombie expression. CD3+ T cells were then identified and CD4+ or CD8+ T cells further profiled. CD29 expression in CD4+ T cells was then profiled and various cytokines such as IFN-γ, CD107a, IL21, TNF-α and IL-4 in CD29 lo vs CD29 hi CD4+ T cells unstimulated, gag and PMA/ionomycin conditions.

Supplementary Figure 6 evaluation of cytokine secretion and cytotoxic molecule expression in cd29lo cd4+ t cells of cart treated siv infected macaques exposed to morphine or saline. Levels of **(A)** ifn-γ **(B)** il-4 **(C)** tnf-α **(D)** cd107a **(E)** mfi of granzyme b **(F)** granzyme b hi **(G)** ifn-γ and tnf-α **(H)** il-21 and ifn-γ expression in cd29lo cd4+ t cells.

Supplementary Figure 7 evaluation of cytotoxic molecules, transcription factors in cart treated siv infected macaues exposed to morphine or saline. Cd3+ t cells were identified from live cells. Cd4+ t cells were then gated out followed by testing for cd29 expression. In unstimulated, gag or pma/ionomycin conditions, percent expression of: the cytotoxic molecule granzyme together with transcription factors like t bet or eomes were evaluated in cd29 hi vs cd29 lo subsets.

Supplementary Table 1 various antibody panels used for the study. Exhaustive list of monoclonal antibodies with insights into specificity, fluorochrome tagged, ab clone, vendor/source together with volumes/test used.
