# Supplementary material for: Diminished Peripheral CD29hi Cytotoxic CD4+ T Cells Are Associated With Deleterious Effects During SIV Infection
Source: Front Immunol. 2021 Oct 13;12:734871. doi: 10.3389/fimmu.2021.734871 (PMC8548621; doi:10.3389/fimmu.2021.734871)
Supplement: Supplementary Table 1 — various antibody panels used for the study. Exhaustive list of monoclonal antibodies with insights into specificity, fluorochrome tagged, ab clone, vendor/source together with volumes/test used. [file Table_1.docx]

| **Specificity** | **Flourochrome** | **Ab clone** | **Vendor** | **Catalogue No** | **Vol/ test** |
| --- | --- | --- | --- | --- | --- |
| CD3 | APC-H7 | SK7 | BD-Pharminogen | 557832 | 2.5 ul |
| CD8 | BUV805 | SK1 | BD-biosciences | 564912 | 0.625 ul |
| TCRγ/δ | FITC | B1 | Biolegend | 331208 | 1.5 ul |
| CD4 | PE-CF594 | L200 | BD-biosciences | 562402 | 2.0 ul |
| CD11a/ LFA-1 | APC | HI111 | BD-biosciences | 559875 | 10 ul |
| CD11b | PE/Cy7 | M1/70 | Biolegend | 101216 | 2.0 ul |
| CD40L (CD154) | Pacific Blue | 24-31 | Biolegend | 310820 | 2.0 ul |
| CX3CR1 | PE | K0124E1 | Biolegend | 355704 | 2.0 ul |
| CD29 | PE-Cy5.5 | MAR4 | BD-biosciences | 559882 | 10 ul |
| Zombie Aqua (Live Dead) | BV510 |  | Biolegend | 423102 | 1:1000 |

| **Specificity** | **Flourochrome** | **Ab clone** | **Vendor** | **Catalogue No** | **Vol/ test** |
| --- | --- | --- | --- | --- | --- |
| CD3 | APC-H7 | SK7 | BD-Pharminogen | 557832 | 2.5 ul |
| CD8 | BUV805 | SK1 | BD-biosciences | 564912 | 0.625 ul |
| CD4 | FITC | M-T477 | BD-biosciences | 556615 | 1.5 ul |
| HLA-DR | PE-Texas Red | TU36 | Life Technologies | MHLDR17 | 2.0 ul |
| CD161 | APC | [HP-3G10](https://www.biolegend.com/ja-jp/search-results?Clone=HP-3G10) | Bio legend | 339912 | 2.0 ul |
| Vα7.2 Jα | PE/Cy7 | 6B11 | Bio legend | 342912 | 2.0 ul |
| CD56 | BV450 | B159 | BD-biosciences | 560360 | 2.0 ul |
| NKG2A | PE | Z199 | Beckman Coulter | PN IM3291U | 2.0 ul |
| CD29 | PE-Cy5.5 | MAR4 | BD-biosciences | 559882 | 10 ul |
| CD16 | BV711 | 3G8 | BD-biosciences | 563127 | 2.0 ul |
| Zombie Aqua (Live Dead) | BV510 |  | Bio legend | 423102 | 1:1000 |
| CD27 | BUV395 | M-T271 | BD-biosciences | 740291 | 0.625 ul |

**Panel for Integrin expression**

**Panel for lineage markers**

| **Specificity** | **Flourochrome** | **Ab clone** | **Vendor** | **Catalogue No** | **Vol/ test** |
| --- | --- | --- | --- | --- | --- |
| CD3 | BV605 | SP34-2 | BD-Horizon | 562994 | 1.25ul |
| CD8 | BUV805 | SK1 | BD-biosciences | 564912 | 0.625 ul |
| Perforin | FITC | δG9 | BD Pharmingen™ | 556577 | 10 ul |
| CD4 | AF700 | L200 | BD-biosciences | 560836 | 2.0 ul |
| Granzyme B | APC | GB12 | Invitrogen | **MHGB05** | 2.5 ul |
| NKG2A | PE/Cy7 | Z199 | Beckman Coulter | B10246 | 2.0 ul |
| T bet | Eflour 450 | eBio4B10 (4B10) | BD-biosciences | 48-5825-82 | 2.5 ul |
| Eomes | PE-eflour 610 | WD1928 | Invitrogen | 61-4877-42 | 2.5 ul |
| NKG2D | PE | 0N72 | Beckman Coulter | A08934 | 2.0 ul |
| CD29 | PE-Cy5.5 | MAR4 | BD-biosciences | 559882 | 10 ul |
| Near IR (Live Dead) | APC H7 |  | Life Technologies | L34975 | 1:1000 |
| CD27 | BUV395 | M-T271 | BD-biosciences | 740291 | 0.625 ul |

**Cytotoxicity panel**

| **Specificity** | **Flourochrome** | **Ab clone** | **Vendor** | **Catalogue No** | **Vol/ test** |
| --- | --- | --- | --- | --- | --- |
| CD3 | APC-H7 | SK7 | BD Pharmingen™ | 557832 | 2.5 ul |
| CD8 | BUV805 | SK1 | BD-biosciences | 564912 | 0.625 ul |
| CD107a | FITC | H4A3 | BD Pharmingen™ | 555800 | 10 ul |
| CD4 | PE-CF594 | L200 | BD-biosciences | 562402 | 2.0 ul |
| IL-21 | APC | 3A3-N2.1 | BD-biosciences | 560493 | 10 ul |
| TNFα | PE/Cy7 | MAb11 | BD Pharmingen™ | 557647 | 2.5 ul |
| IL-4 | BV421 | MP4-25D2 | BD-biosciences | 564110 | 2.0 ul |
| IFN gamma | PE | B27 | Beckman Coulter | 559327 | 10 ul |
| CD29 | PE-Cy5.5 | MAR4 | BD-biosciences | 559882 | 10 ul |
| Zombie Aqua (Live Dead) | BV510 |  | Bio legend | 423102 | 1:1000 |

**Cytokine secretion**

| **Specificity** | **Vendor** | **Catalogue No** | **Working solution** |
| --- | --- | --- | --- |
| Trypan Blue Stain (0.4%) | Invitrogen | T10282 | 1:1 |
| PMA/ ionomycin cell stimulation cocktail | eBiosciences/ Invitrogen | 00-4975-93 | 1:1000 |
| Anti Hu FC Binding Receptor | Invitrogen | 14-9161-73 | 20 ul/ sample |
| BD Fast Immune  CD28/CD49d | BD-biosciences | 347690 | 1:20 |
| Monensin solution | Bio legend | 420701 | 1:1000 |
| BD golgi plug | BD biosciences | 51-2301KZ | 1:1000 |
| Fix/perm concentrate  (4x) | TONBO biosciences | TNB-1020-L050 | (1x) |
| FoxP3 Transcription factor | TONBO biosciences | TNB-1022-L160 | (1x) |
| BD permwash buffer (10x) | BD-biosciences | 51-209KZ | (1x) |
| Pen Strep (100 X) | Gibco | 15140-122 | (1x) |
| Hepes (1M) | Gibco | 15630-080 | (10 mM) |
| 0.5M EDTA | Gibco | 15575-038 | 20Mm EDTA |
| GlutaMAX (100X) | Gibco | 35050-061 | (1x) |
| RPMI Medium 1640 (1x) | Gibco | 21870-076 | (1x) |
| Phosphate Buffer Saline | Cytiva | SH30256.02 | (1x) |
| Ultra-Comp eBeads | Invitrogen | 01-2222-42 | 1 drop/ test |
| SIV Mac 239 gag | JPt/ NIH AIDS reagent program | 6204 | 2ug/ml |

**Miscellaneous reagents**

| **Reagent** | **Stock Concentration** | **Intermediate Dilution** | **Final Concentration** |
| --- | --- | --- | --- |
| phorbol12-myristate13 acetate (PMA) | 1 mg/mL in  (store in aliquots at -20C) | 1:500 in PBS | 20 ng/ml |
| Gag Peptide mixes (ARP-6204) | 0.5-1 mg/mL/pep in DMSO (store in aliquots at -20C) | 1:10 in PBS | 1 μg/mL/peptide  (1:50 ) |
